# Liquid–liquid equilibrium measurements and computational study of salt–polymer aqueous two phase system for extraction of analgesic drugs

**DOI:** 10.1038/s41598-022-18122-x

**Published:** 2022-08-16

**Authors:** Fariba Ghaffari, Mohammad Khorsandi, Hemayat Shekaari, Mohammed Taghi Zafarani-Moattar

**Affiliations:** Department of Physical Chemistry, University of Tabriz, Tabriz, Iran

**Keywords:** Chemistry, Chemical engineering, Physical chemistry, Theoretical chemistry

## Abstract

In recent decades, aqueous two phase systems have gained a lot of attention for extraction of different materials. In this work, an aqueous two phase system was made by polyethylene glycol 600 and potassium hydroxide and phase diagram were determined for this system. The experimental binodal data were described using two empirical nonlinear three parameter expressions developed by Merchuk and Zafarani-Moattar. The consistency of the experimental tie-line data was determined by utilizing the Othmer-Tobias, Bancraft, and Setschenow correlations. Also, the extraction of two analgesic drugs, namely ibuprofen and acetaminophen were investigated by the mentioned ATPS. For this purpose, partition coefficients and extraction efficiencies of each drug were calculated. The trend of extraction efficiencies indicated that the responsibility of extraction of the mentioned drugs into the polymer-rich top phase is related to their hydrophobicity. The Diamond-Hsu equation and its modified version were used to correlate the drugs experimental partition coefficients. Furthermore, the interaction of mentioned drugs with polyethylene glycol was investigated employing quantum computing techniques based upon density functional theory (DFT). These results were in good agreement with the trend of extraction efficiencies of studied drugs.

## Introduction

Many types of biotechnological interest molecules, such as proteins, peptides, and other tiny bioactive compounds, could be extracted and recovered using systems comprising two liquid phases at certain critical concentrations^1,2^. To retain the structure and function of such compounds, biphasic systems with two water-rich phases are preferable to those with one aqueous and one organic phase. In fact, their high water content suggests greater biocompatibility and lower interfacial tension values, hence decreasing biomolecule destruction during the transfer from one phase to the other. These liquid–liquid systems are called aqueous two phase systems (ATPSs). An ATPS is mostly composed of water and non-volatile components. The components could be two structurally distinct hydrophilic polymers or one polymer and one inorganic salt.

In recent years many research groups have focused on the measurement of new equilibrium data for aqueous polymer + salt systems^3–7^. Some of the polymers used in the ATPS partitioning studies, such as polyethylene glycol (PEG), are nonionic hydrophilic polymers. Due to the non-toxic nature of PEG, it has many applications in various fields, such as cosmetics, food, and pharmaceutical products. PEG due to its availability is commonly used to create ATPSs for partitioning processes and precipitation of bio-macromolecules^1,8,9^. This polymer with kosmotropic (i.e. water structure) salts can be used to form ATPSs. There are some experimental liquid–liquid equilibria (LLE) data for ATPSs composed of this polymer and sodium hydroxide^9–11^. But there is no information for ATPs containing PEG and potassium hydroxide.

So in this work, an ATPS composed of polyethylene glycol with a molar mass of 600 g·mol-1 (PEG_600_), and potassium hydrochloride (KOH), was studied at 298.15 K and atmospheric pressure (≈85 kPa). For mentioned ATPS, the binodal and tie-line values were determined. Merchuk^12^ and Zafarani- Moattar et al.^13^ equations were used to fit the binodal data. The consistency of the measured LLE data was tested using the Othmer-Tobias^14^, Bancraft^15^, and Setschenow^16^ equations.

Also, the application of this ATPS was studied for partitioning of ibuprofen and acetaminophen as widely used analgesic drugs. The partitioning coefficients, *K*, and the corresponding extraction efficiency, *EE* %, at different tie-lines were calculated to the investigation of effect of drug nature on the separation of mentioned drugs. The Diamond-Hsu^17^ equation and its modified form were used to fit the partitioning coefficient values.

Finally, this paper has investigated the aspects of binary systems, including PEG + Ibuprofen, PEG + Acetaminophen, KOH + Ibuprofen, and KOH + Acetaminophen. In this case, the geometry optimization of all possible configurations of systems incorporating these systems was computed utilizing the density functional theory (DFT) approach. From an experimental and comprehensive theoretical standpoint, including natural bond orbital (NBO), quantum theory of atoms in molecules (QTAIM), and non-covalent interaction (NCI) studies, have been applied to elucidate the posture of extraction and phase separation of these pharmaceutical compounds.

## Experimental section

### Materials

All data corresponding to the chemicals used in this study have been shown in Table 1. The poly(ethylene glycol) with a molar mass of 600 g mol^−1^ and potassium hydroxide with a minimum purity of 99% were purchased from Merck. Ibuprofen and acetaminophen (> 99.5 wt%) were obtained from Danna pharmaceutical company (Iran). All materials were used without further purification and double distilled and deionized water was used in preparation of solutions.

**Table 1:** Descriptions of the used chemicals.

Material	Provenance	CAS number	Purity (mass fraction)	Purification method	Water content^a^ (mass fraction)
PEG_600_	Fluka	25322-68-3	> 0.990	None	0.0020
Potassium hydroxide	Merck	1310-58-3	> 0.990	None	0.0001
Ibuprofen	Danna. Iran	–	> 0.995	None	0.0001
Acetaminophen	Danna. Iran	–	> 0.995	None	0.0001

^a^Determined by Karl–Fischer method.

### Methods

#### Determination of phase diagrams and tie-lines

The cloud point titration method^18,19^ was used to create the phase diagrams at 298 K and under atmospheric pressure (85 kPa). In this method dropwise additions of potassium hydroxide aqueous solution to PEG_600_ solution were repeated until a cloudy solution (biphasic region) was detected, then water was added dropwise until a clear and monophasic region was observed. To determine the binodal curves, aqueous solutions of PEG_600_ at 60 wt% and aqueous solutions of potassium hydroxide at 50 wt% were used. Throughout the experiment, the solution was constantly stirred. During the preparation of the solutions water contents of chemicals, which were measured by Karl Fischer coulometer (Metrohm 751 GPD) were taken into account. An analytical balance (Shimadzu, 321-34553, Shimadzu Co., Japan) with a precision of ± 1∙10^–7^ kg was used to determine the mass fraction of the components. The maximum uncertainty in determining the mass fraction of polymer and salt was found to be 0.002.

Five different solutions of studied ATPS consist of {PEG_600_ + KOH + water} were prepared to determination of tie-lines. For each tie-line, a mixture of salt, polymer and water at the biphasic region was gravimetrically prepared within ± 10^–7^ kg. These solutions were vigorously stirred for 30 min, centrifuged and placed in water bath at 298 K to reach equilibrium. The compositions of coexisting phases were analytically determined. After separation of the two phases, the concentrations of KOH in the top and bottom phases were determined by flame photometer (JENVEY model PFP7, England). The concentration of PEG in both phases was determined by refractive index measurements^20^ performed at T = 298.15 K using a refractometer (ATAGO DR-A1, Japan) with a precision of ± 0.0001. The uncertainty in refractive index measurement is ± 0.0002. For dilute aqueous solutions containing a polymer and a salt, the relation between the refractive index, *n*_*D*_, and the mass fractions of polymer, *w*_*p*_, and salt, *w*_*s*_ is given by:

1 $$ n_{D} = n_{0} + a_{p} w_{p} + a_{s} w_{s} $$

here *n*_*0*_ is the refractive index of pure water for which the value 1.3325 at T = 298.15 K was obtained. The values of constants *a*_*p*_, and *a*_*s*_ corresponding to polymer, and salt respectively were obtained from the linear calibration plots of the corresponding refractive index of the solutions diluted in mass fraction range (C Range(w/w)) as showed in Table S1. These constants together with the coefficient of determination values, *R*^2^, are reported in Table S1.

#### Partition coefficient and the corresponding extraction efficiency

To assess the performance of the investigated ATPS in terms of drug partitioning and extraction efficiency, the five mixture compositions (reported in Table 2) were chosen based on the phase diagrams determined before for {PEG_600_ + KOH + H_2_O} system. These mixtures had similar overall compositions used to obtain tie-lines. The prepared mixtures were vigorously stirred for 30 min before being placed in a water bath at the desired temperature. After observation of phase separation 1 mL of each phase was carefully separated, mixed again and then 0.002 mass fractions of drugs (ibuprofen and acetaminophen) was added to each sample. The samples were centrifuged at 2,000 rpm for 10 min and kept in a water bath for 24 h to ensure complete phase separation and equilibrium. Finally, after careful separation of phases in equilibrium, the concentrations of acetaminophen in both phases were determined by using a UV spectroscopy using a spectrophotometer mentioned above at the wavelengths of (284, 261 and 303) nm. Also, for analysis of ibuprofen a fluorescence spectrophotometer (F-4500, Hitachi, Tokyo, Japan) was used. To avoid interference from the phase components, the samples were diluted and analyzed against the blanks containing the same phase components but without drugs^21^.

**Table 2:** Experimental tie-line data of {PEG_600_ + KOH + H_2_O} systems at 298.15 K and atmospheric pressure (≈ 85 kPa).

Overall composition (wt%)		PEG-rich phase composition (wt%)		Salt-rich phase composition (wt%)		
[KOH]	[PEG]	[KOH]	[PPG]	[KOH]	[PPG]	*TLL*
17.58	28.98	9.44	61.42	22.43	10.47	52.58
18.95	29.09	9.34	63.92	24.13	10.05	55.86
20.48	29.05	9.43	65.94	25.90	9.86	58.45
21.91	29.11	9.43	68.52	27.51	9.26	61.96
23.13	29.12	9.45	71.32	29.79	8.41	66.12

The standard uncertainty for weight percent of each component is 0.8.

The partitioning coefficient, *K*, and extraction efficiency, *EE* %, were calculated respectively by Eqs. (2) and (3) as below:

2 $$ K = \frac{{wtop_{drug} }}{{wbot_{drug} }} $$

3 $$ EE\% = \frac{K}{K + 1} \times 100 $$

here $wtop_{drug}$ and $wbot_{drug}$ are the mass fractions of drug in the top and bottom phases respectively.

#### Determination of the tie-lines length

The tie-line length (TLL) has unit of %w/w, same as the component concentrations. The TLL can be related to the equilibrium phase composition as follows:

4 $$ TLL = \sqrt {\left( {w_{p}^{top} - w_{p}^{bot} } \right)^{2} + \left( {w_{s}^{top} - w_{s}^{bot} } \right)^{2} } $$

where $w_{p}$ and $w_{s}$ are equilibrium compositions of PEG_600_, and salt, respectively. Superscripts “bot” and “top” designate the PPG_600_-rich phase (top phase) and salt-rich phase (bottom phase), respectively.

## Computational

The interaction of Ibuprofen and acetaminophen with PEG was investigated employing quantum computing techniques based upon density functional theory (DFT). Noteworthy to mention that the investigation of intramolecular interactions in systems including pharmaceutical compounds with KOH and PEG indicates that drugs with PEG have stronger intermolecular interactions, including hydrogen interactions toward drugs with KOH, which would be the results of extraction and separation of these compounds confirm this pattern. In this regard, a computational investigation of binary systems comprising drugs and PEG is being investigated in this research effort. All electrical calculations have been performed using the Gaussian 09 set of tools. The natural bond orbitals (NBO), quantum theory of atoms in molecules (QTAIM), non-covalent interaction (NCI), and energetic parameters were estimated following the optimization of the ibuprofen, acetaminophen and PEG structures. The NBO function in the Gaussian 09 software package was used for all NBO computations. QTAIM investigations were also carried out using Multiwfn software.

The B3LYP functional, a hybrid functional with reliable and consistent performance in investigating diverse compounds' electronic structures and characteristics, was utilized to improve the systems, including Ibuprofen, acetaminophen and PEG. In addition, Grimm's third version of the empirical correction with the Bijerlin effect was included to account for the effect dispersion effect, and the B3LYP-D3 (BJ) functional was employed to optimize the structures. The Multiwfn software has been employed to optimize the most stable structures, further analyzed using the QTAIM approach^22^.

Based on the sign of the eigenvalues of the diagonalited hessian matrix of the Laplacian of electron density ($\nabla^{2} \rho (r)$), the critical points (i.e. where the divergence of $\rho$ is equal to zero) were calculated and used to characterize the critical points as the atom, bond, ring, and cage critical points in QTAIM analysis^23,24^. Bond critical points (BCP) are nearly (3,−1) critical points that are used to detect and characterize potential bond interactions. In this analysis, different factors can be used to decide about the magnitude of $\rho$ the magnitude and the sign of $\nabla^{2} \rho (r)$, the ratio of kinetic (G(r)) to potential energy $(\left| {V(r)} \right|)$ and the value of the total energy (H(r)) and also the ellipticity of the electron density ($\varepsilon_{{}}$) are some of the applicable parameters.

Because the B3LYP functional is somewhat unsuccessful in determining weak van der Walls interactions due to its asymptotic behavior, single-point energy calculations on optimized structures were performed using other functional such as M06-2X and WB97X-D3 to explore the interaction energies more consistently.

The values of total interaction energy were calculated using Eq. (5):

5 $$ E_{{\text{int}}} = E_{AB} - E_{A} - E_{B} + E_{BSSE} $$

which *E*_int_ represents the total interaction of the (Drugs-PEG) systems, E(A) denotes the Drugs energy, and E(B) includes the energy of systems with PEG. The compounds PEG + Ibo and PEG + ACE with the most energetically stable structure were selected. Furthermore, at the B3LYP/6-311 G (d) level of theory, some quantitative conceptual parameters of natural bond orbitals (NBO), quantum theory of atoms in molecules (QTAIM), and Reduced Density Gradient (RDG) analysis were used to confirm the presence of intramolecular interaction, including hydrogen bonds. The Wiberg bond index (WBI), which is defined using the following equation, is another idea that may be used to analyze bond orders^25^:

6 $$ WBI = \sum\limits_{k} {p_{jk}^{2} = 2p_{jj} - p_{jj}^{2} } $$

The density matrix elements are denoted by pjk, whereas the atomic orbital charge density is shown by pjj. Furthermore, the energy of donor–acceptor interactions in the NBO basis might be estimated using the second-order perturbation technique as follows^26^:

7 $$ \Delta E_{i \to j}^{2} = - 2\frac{{\left\langle {\sigma_{i} \left| {\mathop F\limits^{ \wedge } } \right|\sigma_{j}^{*} } \right\rangle^{2} }}{{\varepsilon_{j*} - \varepsilon {}_{i}}} $$

in which b $\mathop F\limits^{ \wedge }$ is the effective orbital Hamiltonian, and ε_i_ and εj* are defined as $\varepsilon_{i} = \left\langle {\sigma_{i} \left| {\mathop F\limits^{ \wedge } } \right|\sigma_{i} } \right\rangle$ and $\varepsilon_{j*} = \left\langle {\sigma_{j}^{*} \left| {\mathop F\limits^{ \wedge } } \right|\sigma_{j}^{*} } \right\rangle$, respectively.

## Results and discussion

### Phase diagram

**Figure 1:**
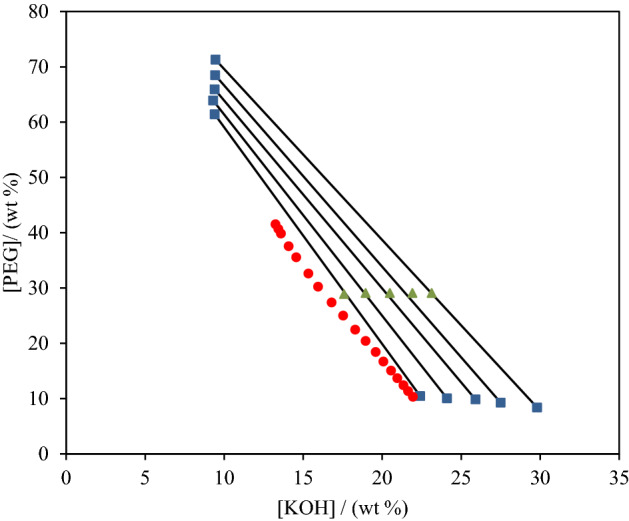
Phases diagram for the system {KOH + PEG_600_ + H_2_O} at 298 K: binodal curve (● red), tie-line overall composition (▲), tie-line phase composition (■).

The phase diagrams of studied ATPS composed of PEG_600_, KOH and water at 298.15 K are plotted in Fig. 1, and the experimental mass fraction data are also listed in Table S2.

The binodal data presented in Table S2 were fitted to the equations presented by Merchuk^12^, Eq. (8), and Zafarani-Moattar et al.^13^, Eq. (9) using a nonlinear least-square regression method.

8 $$ w_{p} = aexp \, \left( {bw_{s}^{0.5} {-} \, cw_{s}^{3} } \right) $$

9 $$ w_{p} = \alpha + \beta \ln (w_{s} ) + \gamma w_{s} $$

In above equations $w_{p}$ and $w_{s}$ are mass fractions of polymer and salt, respectively. The adjustable parameters of Eqs. (5) and (6) {(*a*, *b*, and *c*) and ($\alpha$, $\beta$ and $\gamma$)} together with the corresponding standard deviation, *sd*, are presented in Table S3. The obtained values of *sd* indicate a good performance of both equations in regeneration of binodal data.

### Analysis of tie-lines

The tie-lines together with binodal curves are presented in Fig. 1 for ATPS composed of PEG_600_, KOH, and water. Five experimental tie-lines and the corresponding length (*TLL*) were determined for the mentioned system. The related data are collected in Table 2. It can be seen from this Table, the top phase is always rich in PEG_600_. The majority of bottom phase is composed of higher concentration of salt and water.

#### Tie-line correlation

In this work for the correlation of LLE data of (PEG_600_ + KOH + water) system several models with different thermodynamics basis were used and compared their performances in the correlation of the tie-lines.

##### Othmer-Tobias and Bancraft equations

The correlation equations given by Othmer-Tobias^14^, Eq. (10), and Bancraft^15^, Eq. (11), have been used to correlate the tie-lines composition:

10 $$ \left( {\frac{{1 - w_{p}^{top} }}{{w_{p}^{top} }}} \right) = k\left( {\frac{{1 - w_{s}^{bot} }}{{w_{s}^{bot} }}} \right)^{n} $$

11 $$ \left( {\frac{{w_{w}^{bot} }}{{w_{s}^{bot} }}} \right) = k_{1} \left( {\frac{{w_{w}^{top} }}{{w_{p}^{top} }}} \right)^{r} $$

where $$k$$,$$n$$, $$k_{1}$$, and $$r$$ represented fit parameters. These equations have also been used to assess the reliability of LLE data. Using the tie-line data reported in Table 2, linear dependency of the $$\log ({\raise0.7ex\hbox{${1 - w_{p}^{top} }$} \!\mathord{\left/ {\vphantom {{1 - w_{p}^{top} } {w_{p}^{top} }}}\right.\kern-\nulldelimiterspace} \!\lower0.7ex\hbox{${w_{p}^{top} }$}})$$ plots against $$\log ({\raise0.7ex\hbox{${1 - w_{s}^{bot} }$} \!\mathord{\left/ {\vphantom {{1 - w_{s}^{bot} } {w_{s}^{bot} }}}\right.\kern-\nulldelimiterspace} \!\lower0.7ex\hbox{${w_{s}^{bot} }$}})$$ and $$\log ({\raise0.7ex\hbox{${w_{w}^{bot} }$} \!\mathord{\left/ {\vphantom {{w_{w}^{bot} } {w_{s}^{bot} }}}\right.\kern-\nulldelimiterspace} \!\lower0.7ex\hbox{${w_{s}^{bot} }$}})$$ against $$\log ({\raise0.7ex\hbox{${w_{w}^{top} }$} \!\mathord{\left/ {\vphantom {{w_{w}^{top} } {w_{p}^{top} }}}\right.\kern-\nulldelimiterspace} \!\lower0.7ex\hbox{${w_{p}^{top} }$}})$$ is obtained which indicated an acceptable consistency of the results^14^. The fitting parameters and corresponding standard deviation are shown in Table S4. On the basis of the obtained standard deviations (*sd*), we conclude that Eqs. (10) and (11) can be satisfactorily used to correlate the tie-line data of the investigated system.

##### Setschenow equation

The following Setschenow equation proposed by Hey and coworkers^16^ has also been used for the correlation of tie-line data of studied systems:

12 $$ \ln \left( {\frac{{C_{p}^{top} }}{{C_{p}^{bot} }}} \right) = k_{p} + k_{s} (C_{s}^{bot} - C_{s}^{top} ) $$

in which the $k_{s}$ is the salting-out coefficient, $k_{p}$ is a constant, and $C_{p}$ and $C_{s}$ are the molality of polymer and salt respectively. The parameters of the Eq. (12) which were obtained from the correlation of the experimental LLE data are also given in Table S4 along with the corresponding deviations. According to the *sd* values in Table S4, it could be estimated that correlation with Setschenow equation is satisfactory.

### The performance of salt -PEG ATPS in the partition behavior of drugs

**Figure 2:**
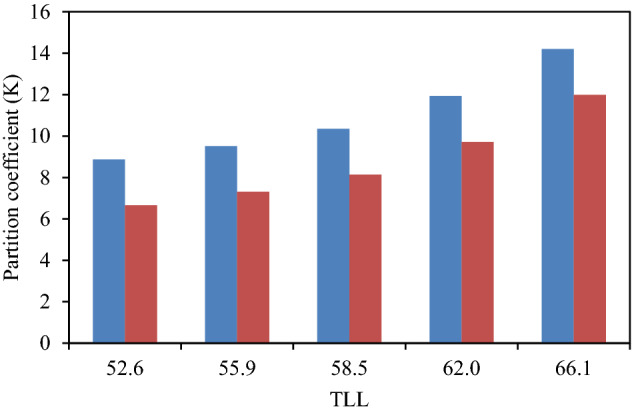
Partition coefficient, *K*, in function of the *TLL* for each studied drug in ATPS {KOH + PEG_600_ + H_2_O}. Ibuprofen  Acetaminophen .

**Figure 3:**
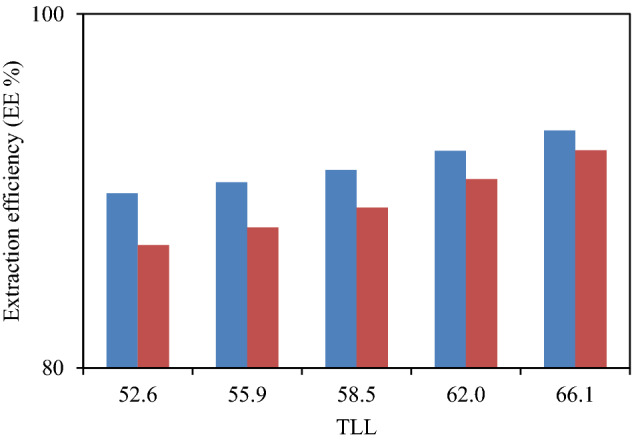
Extraction efficiency, *EE* %, in function of the *TLL* for each studied drug in ATPS {KOH + PEG_600_ + H_2_O}. Ibuprofen Acetaminophen .

The experimental partition coefficients, *K*, and the percentage extraction efficiencies, *EE* %, of investigated drugs (ibuprofen and acetaminophen) are presented in Table 3 and their variation with *TLL* can be seen in Figs. 2 and 3, respectively. The observed trend is as follows: *K* (ibuprofen) > *K* (acetaminophen). It is observed from Table 3 that drug effectively partitioned to the PPG-rich phase (more hydrophobic phase).

**Table 3:** Values of partitioning coefficients, *K*, and extraction efficiency, *EE* %, of drugs for the system {PEG_600_ + KOH + H_2_O} at 298.15 K and atmospheric pressure (≈ 85 kPa).

Overall composition (wt%)		*K*	*EE*%
[KOH]	[PEG]		
**Ibuprofen**			
17.58	28.98	8.87	89.87
18.95	29.09	9.51	90.49
20.48	29.05	10.35	91.19
21.91	29.11	11.93	92.27
23.13	29.12	14.20	93.42
**Acetaminophen**			
17.58	28.98	6.66	86.95
18.95	29.09	7.31	87.95
20.48	29.05	8.14	89.06
21.91	29.11	9.72	90.67
23.13	29.12	11.99	92.30

The standard uncertainties *σ* for temperature, pressure and partitioning coefficient are: *σ* (*T*) = 0.05 K, *σ* (*p*) = 0.5 kPa and *σ* (*K*) = 0.15, respectively.

#### The effect of drug structure on the drug partitioning behavior

The chemical structures and polarities of drug are responsible for the observed trend of the *K* and *EE* % values (Table 3). The studied drugs because of their non-polar properties have favorable interactions with hydrophobic phase (PEG- rich phase), and therefore, partitioning of these drugs to the salt-rich phase occurs significantly less frequently. In fact, the presence of non-polar groups in drug structure increases its tendency to PEG-rich top phase. The obtained high values of *EE* % collected in Table 3 imply that both of the investigated drugs partitioned mostly in the PEG-rich top phase. The observed trend for the drugs *K* values is in good agreement with their hydrophobicity values defined by the logarithm of octanol/water partition coefficient (log *K*_ow_); so that higher log *K*_ow_ values show that the solute have more hydrophobicity and lower tendency to water molecules^18^. The log *K*_ow_ values of drugs are 3.97^27^ and 2.34^28^ for ibuprofen and acetaminophen, respectively. Therefore the drug extraction efficiency is in the order: ibuprofen > acetaminophen. This study and previous one^29^ indicate that the hydrophobicity of drugs can be an important criteria to predict of drug partitioning between two-phases of an ATPS.

#### Correlations of the partition coefficients

For regeneration of the partition coefficient data a model is required; and here, the equation proposed by Diamond-Hsu^17^ (Eq. 13) was used for correlation of experimental partition coefficients values of the studied drugs. We also attempted to improve the quality of the Diamond-Hsu model by adding one extra term to the right hand side of Eq. (13) and refitting the *K* values to the modified polynomial equation (Eq. 14):

13 $$ \ln K = A.\Delta w(PEG_{600} ) + B.\Delta w(PEG_{600} )^{2} $$

14 $$ \ln K = A_{1} + B_{1} .\Delta w(PEG_{600} ) + C_{1} .\Delta w(PEG_{600} )^{2} $$

In above relations, (*A* and *B*) and (*A*_1_, *B*_1_, and *C*_1_) are respectively the adjustable parameters of Eqs. (13) and (14). The symbol $\Delta w(PEG_{600} )$ is used for the mass fraction difference of PEG_600_ in the top and bottom phase.

For the studied drugs the results of fitting experimental partition coefficients values to Eqs. (13) and (14) together with the corresponding standard deviations, *sd* are reported in Table S5. According to the *sd* values in Table S5, it could be estimated that correlation with both of the equations is satisfactory.

### Computational section

#### The optimized structures and energetic parameters

The initial structures of the drugs and PEG have been optimized in various configurations to determine the most stable compounds with the minimum relative energy in binary systems based on PEG + acetaminophen and PEG + ibuprofen, which can be seen in Table 4. Furthermore, the energy of various configurations of these binary systems has been provided in the supplementary information from Tables S6 and S7.

**Table 4:** The energy difference (**Δ***E*_*int*_) of binary systems for the three functional methods.

$\Delta E_{{\text{int}}} = E_{AB} - E_{A} - E_{B} + E_{BSSE}$^a^			
Binary systems			
	B3LYP-D3(BJ)	M06-2x	WB97xd
Acetaminophen-PEG^b^	− 16.59	− 16.84	− 16.90
Ibuprofen-PEG	− 9.44	− 9.28	− 9.63

^a^The E_AB_ indicate the energy of binary systems, The E_A_ demonstrative of the energy of Drugs and The E_B_ demonstrative of the PEG energy in the mentioned systems.

^b^Ace = Acetaminophen, Ibo = Iboprophen, PEG = Poly ethylene glycol.

The various configurations for each binary system (6 and 8) were considered. In this regard, a total of 6 and 8 distinct binary complexes, comprising drugs and PEG, were evaluated and geometrically optimized.

The obtained results show that the PEG + ibuprofen systems have the lowest structural energy (the most negative value) in comparison to the other compounds in all three functional studies, indicating that the investigated drugs + PEG are the most stable in terms of energy and structure.

Besides addition, based on the QTAIM, NBO, and NCI analyses, the most stable acetaminophen + PEG and ibuprofen + PEG complexes were utilized to evaluate.

#### The (QTAIM) analyses

The quantum theory of atoms in molecules (QTAIM) would be another critical and prevalent technique. By employing Multiwfn software, the most stable optimized structures were further evaluated using the QTAIM approach. In investigating the nature of possible intermolecular interactions, the QTAIM was also researched on the studied binary system.

Bader's QTAIM method^30,31^, which uses specific definitions including electron density ($\rho (r)$) and Laplacian of electron density ($\nabla^{2} \rho (r)$), is a sophisticated analysis method for determining the nature of various sorts of interactions^32^. Critical points (i.e., a minimum, maximum, or saddle point in the spatial curvature of ($\rho (r)$) are discovered in QTAIM analysis when the gradient of electron density ($\nabla^{2} \rho (r)$) is identically zero, and the Hessian matrix of ($\rho (r)$) is calculated and utilized to characterize critical points. The eigenvalues ($\lambda_{1}$), ($\lambda_{2}$), and ($\lambda_{3}$) of the diagonalized Hessian matrix would be used to categorize the critical locations (CPs).

To evaluate whether a weak or strong attractive intermolecular or interatomic bonding interaction exists, the presence of a bond critical point (BCP) is crucial^23,24^.

Chemical interactions could be classified into various classes based on the $\rho (r)$ and $\nabla^{2} \rho (r)$ amounts obtained by the QTAIM analysis at the BCP. As a outcomes, a negative $\nabla^{2} \rho (r)$ value and a $\rho (r)$ value more than 0.1 a.u. at a BCP identify the BCP as a shared (covalent) bonding^33^. However, the bonding interactions can be classified as non-covalent, strong ionic, or weak Van der Waals for positive $\nabla^{2} \rho (r)$ values.

The quantum virial theorem^33–35^ could be a significant relationship between energetic QTAIM parameters and P1 at CPs, as evidenced by the following relationship:

15 $$ \frac{1}{4}\nabla^{2} \rho (r) = 2G(r) + V(r) $$

where $V(r)$ are $G(r)$ the potential and kinetic energy densities of electrons, respectively.

Applying Multiwfn software, the most stable optimized structures were further analyzed using the QTAIM method. Table 5 exhibit binary systems including acetaminophen + PEG and ibuprofen + PEG complexes, respectively, based on the above investigations. It is important to note that only the strongest interactions with the highest values of $\rho (r)$ were presented in this table. This table demonstrates that the strong interaction is caused by the H atoms of Ibo and acetaminophen with the –O of PEG interaction. The binary interaction between ibuprofen-PEG and Ace PEG has 0.03 ≤ $\rho (r)$ ≤ 0.05, positive $\nabla^{2} \rho (r)$ and 0.5 ≤ ${{G(r)} \mathord{\left/ {\vphantom {{G(r)} {|V(r)|}}} \right. \kern-\nulldelimiterspace} {|V(r)|}}$ ≤ 1 and negative values, indicating that all interactions are mixed ionic-covalent. As an outcome, the results for ibuprofen-PEG and acetaminophen-PEG were reported as 0.0.0395 and 0.0342 kg/m^3^, respectively, as shown in Table 5.

**Table 5:** The AIM topological parameters, including electron density (*ρ*), Laplacian of electron density (∇^2^*ρ*(r)), the kinetic electron density *G*(r), potential electron density *V*(r), eigenvalues of Hessian matrix (*λ*) and bond ellipticity index (*ε*) of binary systems containing drugs and PEG.

System	Interaction	$\rho (r)$	$\nabla^{2} \rho (r)$	$H(r)$	$G(r)$	$V(r)$	${{G(r)} \mathord{\left/ {\vphantom {{G(r)} {|V(r)|}}} \right. \kern-\nulldelimiterspace} {|V(r)|}}$	$\lambda_{1}$	$\lambda_{2}$	$\lambda_{3}$	$\varepsilon$
Ibuprofen_PEG	H(Ibo)…O(EG)	0.0395	0.1182	− 0.0024	0.0319	− 0.0343	0.9300	− 0.021	0.2401	− 0.0598	0.0373
Acetaminophen_PEG	H (Ace)…O(EG)	0.0342	0.1214	0.0003	0.0300	− 0.0297	1.0101	0.0056	− 0.0507	− 0.0535	0.0543

**Figure 4:**
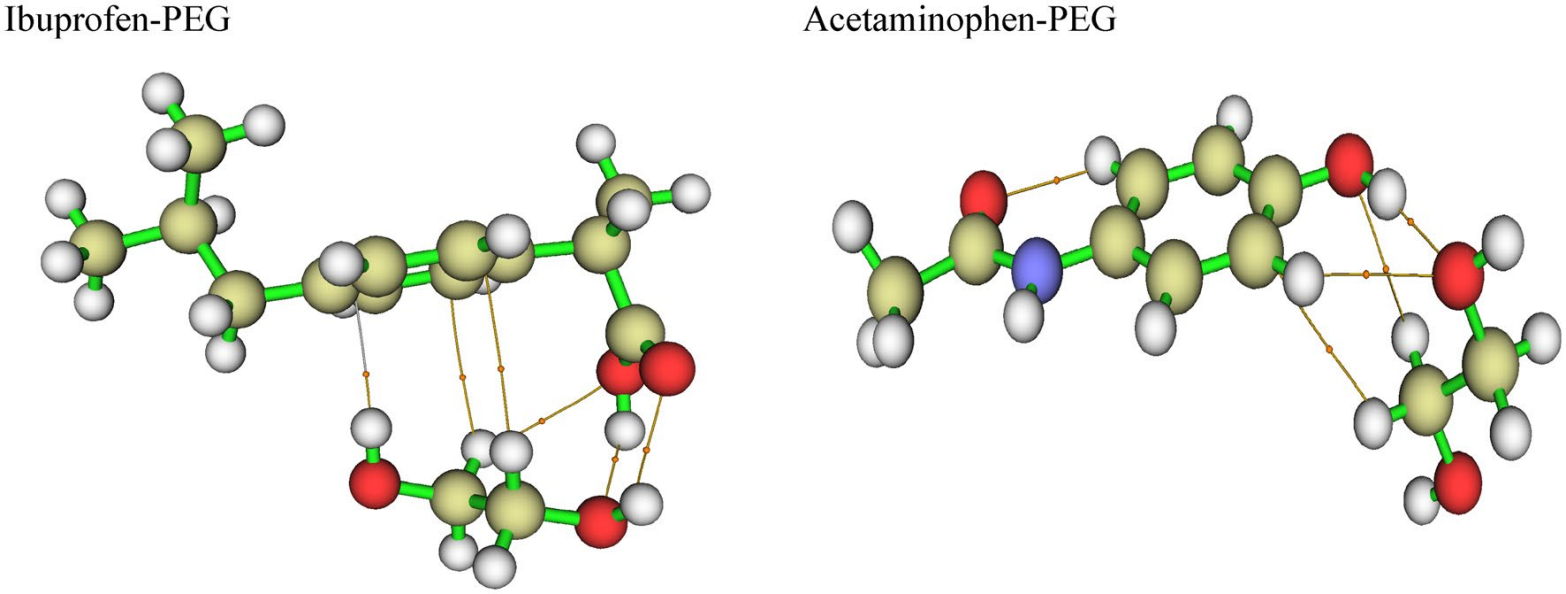
Schematic drawing of binary systems containing Ibuprofen-PEG and Acetaminophen-PEG showing its bond paths.

Additionally, the values are also used to determine the stability of the intramolecular interaction, with $\varepsilon \langle 1$ indicating the most stable structure. In binary systems, the $\varepsilon$ values for ibuprofen-PEG and Acetaminophen-PEG, for instance, have been reported as 0.0373 and 0.0543, respectively. The obtained ${{G(r)} \mathord{\left/ {\vphantom {{G(r)} {|V(r)|}}} \right. \kern-\nulldelimiterspace} {|V(r)|}}$ values are shown in Table 5 and Fig. 4, reported as 0.9300 and 1.0101 for the ibuprofen-PEG and acetaminophen-PEG, respectively.

#### The (NBO), (WBI) values and (N.C.I) analyses

Weinhold et al.^36^ outline the scope of the natural bond orbital (NBO) analysis applied in this work. NBO methods have a mathematical and historical background that could be discovered elsewhere. The second-order perturbation theory defined H-bond strength as delocalization energy E2 (e.g., electron transfer from donor to acceptor orbital) because of the NBO conception. The most stable structures of binary systems were also applied to NBO analysis, with the outcomes presented in Table 6 for some donner-acceptor interactions. It is noteworthy that the interactions with the E^2^ values are included in Table 6. In all circumstances, the most significant interaction is due to the transfer of lone pair electrons (LP) from the π orbitals of O of drugs to the antibonding H–O of PEG substances, as seen in this table. According to Table 6, the ibuprofen-PEG with the highest E^2^ values is the most stable structure compared to the other binary systems. Second-order perturbation energies (E^2^) for ibuprofen-PEG. For instance, the data has been reported as being of the order of 16.12 kcal/mol for the ibuprofen-PEG binary system. The natural charge (N.C.I) and Wiberg bond indices (WBI) values were also employed to investigate the interactions further. Furthermore, the natural charge (N.C.I) and Wiberg bond indices (WBI) values were utilized to evaluate the interactions further. The Table 7 indicates the values of the WBI (bond length (re) and Wiberg bond index of the most significant interactions), which represents the strength of intermolecular interactions between the drugs and PEG in the researched configurations^34,37,38^. The values of WBI are in the region of strong non-covalent interactions, according to the reported data in Table 7. Furthermore, the findings of the NCI analysis are as follows: This investigation utilized NCI analysis to investigate the weak non-covalent interactions outlined in the QTAIM and NBO sections.

**Table 6:** The donor–acceptor NBO interactions and second order perturbation energies (E^2^) for binary systems containing drugs and PEG.

System	Donor NBO (**i**)	Acceptor NBO (**j**)	**E**^2^ (kcal/mol)
Ibuprofen-PEG	LP(O)	BD*(O–H)	16.12
	LP(O)	BD*(O–H)	2.05
Acetaminophen-PEG	LP(O)	BD*(O–H)	12.37
	LP(O)	BD*(O–H)	2.47

**Table 7:** The Wiberg bond order index (WBI), and the smallest interatomic distances (*r*_e_ (Å)) between of binary systems containing drugs and PEG.

System	Interaction	r (Å)	WBI
Acetaminophen-PEG	O…H	1.8060	0.0464
Ibuprofen-PEG	O…H	2.1526	0.0174

The reduced density gradient (RDG) analysis is a method for visualizing and evaluating noncovalent interactions in real space, including as van der Waals contacts, hydrogen bonds, and steric effects, utilizing electron density and its derivatives^39^. One of the other descriptor for evaluating intermolecular interactions appears to be the reduced density gradient (RDG), which is defined as follows^39,40^:

16 $$ RDG = \frac{1}{{2(3\pi^{2} )^{{{\raise0.7ex\hbox{$1$} \!\mathord{\left/ {\vphantom {1 3}}\right.\kern-\nulldelimiterspace} \!\lower0.7ex\hbox{$3$}}}} }}\frac{{\left| {\nabla \rho (r)} \right|}}{{\rho (r)^{{{\raise0.7ex\hbox{$4$} \!\mathord{\left/ {\vphantom {4 3}}\right.\kern-\nulldelimiterspace} \!\lower0.7ex\hbox{$3$}}}} }} $$

Realizing that the strength of the interaction illustrated the good correlation with $sign\lambda_{2} (r)\rho (r)$, a scatter plot of the RDG as a function of $sign\lambda_{2} (r)\rho (r)$ could be applied to recognize the nature of the polymer and drugs interaction.

**Figure 5:**
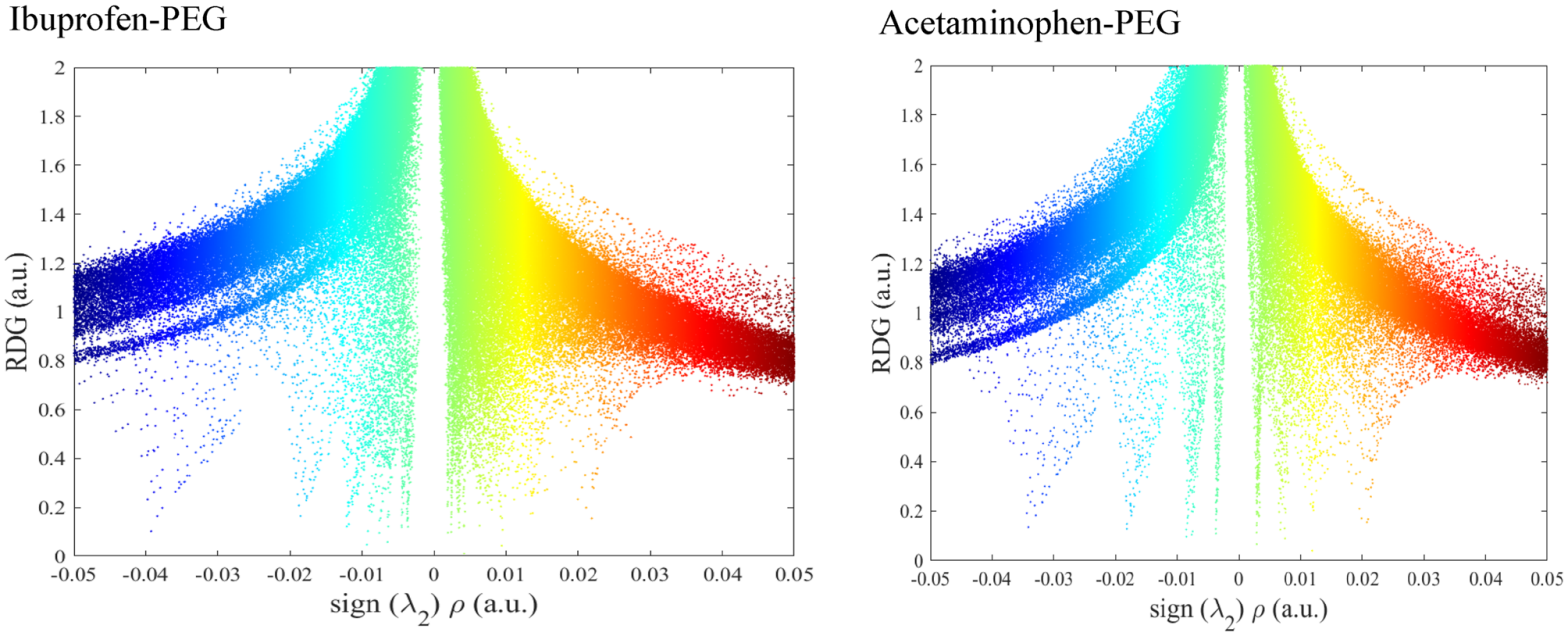
Reduced density gradient versus sign (λ_2_)$\rho$(a.u.) scatter plot of binary systems containing Ibuprofen-PEG and Acetaminophen-PEG.

NCI analysis results in this respect the scatter plot of RDG values were calculated using Multiwfn software and the RDGs versus sign($\lambda_{2}$)$\rho (r)$ were plotted. The results were illustrated for binary complexes in Fig. 5. The existence of narrow spikes in the region of $\sin \lambda (r)\langle 0$, for is surfaces of RDG ˂0.5, the existence of reveals strong non-covalent H–O and hydrogen bonding in all cases. The results also are in full agreement with QTAIM results, the Van der Waals interaction (i.e. where $\rho \approx 0$,$\nabla^{2} \rho \approx 0$ ) and the repulsive steric effects (i.e. where $\lambda_{2} (r)\rangle 0$). This conclusion can also be made from QTAIM and NBO analysis, where in both cases the antisymmetry of the similar interactions is obvious.

## Conclusions

Phase diagrams of an ATPS composed of polyethylene glycol with a molar mass of 600 g mol^−1^, and potassium hydroxide at 298.15 K were studied. The experimental binodal values were satisfactorily adjusted by Merchuk and Zafarani-Moattar et al. two semi-empirical equations. To correlation of tie-lines in the studied system various equations (Othmer-Tobias, Bancraft and Setschenow) were used. Based on the obtained outcomes, it was determined that the performances of all the analyzed equations in the correlation are satisfactory. Additionally, partition coefficient and extraction efficiency for two drugs namely ibuprofen and acetaminophen on the studied ATP were obtained. The trend of the extraction efficiency showed that these drugs due to their hydrophobic properties preferentially partition into the top phase (polymer-rich).

Finally, density functional theory (DFT), QTAIM and NBO calculations were employed to study binary systems' intramolecular and structural properties. Different kinds of DFT functional, including B3LYP-D3 (BJ), M062X and WB97X-D3 combined with two 6-311G (d,p) basis sets were used. The formation energies confirmed that B3LYP-D3 (BJ) is the most suitable functional group among the other functional groups. Also, it could be seen that the ibuprofen-PEG is more stable than the acetaminophen-PEG. The interactions with the highest values of E^2^ obtained from NBO analysis showed the most significant interaction in all cases is due to the donation of the lone pair electrons (LP) from π orbitals of O to the antibonding H of PEG. QTAIM topology analysis showed the positive values for the Laplacian of electron density, which confirms the strong interactions are due to the interaction of the O atom of drugs with H (–O) of PEG. These changes are more intensive in the ibuprofen-PEG binary system than in acetaminophen-PEG. Since the interaction ibuprofen-PEG could change the intermolecular interaction trends more than acetaminophen-PEG, it could be more beneficial for drug separation in the pharmaceutical and chemical industries.

## Supplementary Information

Supplementary Information.
