# Supplementary material for: Liquid–liquid equilibrium measurements and computational study of salt–polymer aqueous two phase system for extraction of analgesic drugs
Source: Sci Rep. 2022 Aug 16;12:13848. doi: 10.1038/s41598-022-18122-x (PMC9381745; doi:10.1038/s41598-022-18122-x)
Supplement: Supplementary file 1 — Supplementary Information. [file 41598_2022_18122_MOESM1_ESM.docx]

Supporting Information

**Liquid-liquid equilibrium measurements and computational study of salt-polymer aqueous two phase system for extraction of analgesic drugs**

Fariba Ghaffari^*^, Mohammad Khorsandi, Hemayat Shekaari, Mohammed Taghi Zafarani-Moattar

*Department of Physical Chemistry, University of Tabriz, Tabriz, Iran*

^*^Corresponding author. Tel: +98 4133393094.

E-mail addresses: faribaghaffari2129@gmail.com.

**Table S1**

Values of the parameters of Eq. 1, *a*_m_, for ABS containing {KOH (s) +PEG_600_ (p) + water (w)} system.

| Material | Constant | Value | C *Range(w/w)* | *^a^R^2^* |
| --- | --- | --- | --- | --- |
| KOH | *a_c_* | 0.1452 | 0 to 0.08 | 0.9989 |
| PEG_600_ | *a_p_* | 0.1323 | 0 to 0.10 | 0.9993 |

*^a^* where, *R^2^,* represented respective correlation coefficient value of the linear calibration plot of the refractive index against mass fraction of potassium hydroxide and polymer at the mass fraction range (*C range*) of each material.

**Table S2**

Experimental binodal data of mass fraction (wt %) for the systems containing {ChCl: sucrose ($w_{NADES}$) + PPG_400_ ($w_{p}$) + H_2_O} at 298.15 K and atmospheric pressure (≈ 85 kPa).*^a^*

| $w_{s}$ | $w_{p}$ |
| --- | --- |
| | 13.25 | | --- | | 13.43 | | 13.59 | | 14.08 | | 14.56 | | 15.33 | | 15.94 | | 16.80 | | 17.53 | | 18.29 | | 18.95 | | 19.59 | | 20.07 | | 20.56 | | 20.95 | | 21.35 | | 21.64 | | 21.94 | | | 41.54 | | --- | | 40.68 | | 39.86 | | 37.55 | | 35.57 | | 32.63 | | 30.25 | | 27.38 | | 25.01 | | 22.48 | | 20.44 | | 18.44 | | 16.70 | | 15.06 | | 13.69 | | 12.42 | | 11.35 | | 10.32 | |

*^a^* The standard uncertainties *σ* for temperature, pressure, and mass fraction are: *σ* (*T*) = 0.05 K; *σ* (*p*) = 0.5 kPa; and *σ* (*w_i_*) = 0.005, respectively.

**Table S3**

The parameters values of Eq. 8, (*a, b, c*), and Eq. 9, (*α, β, 𝛾*) for {PEG_600_ + KOH + H_2_O} systems at 298.15 K.

| Merchuk (Eq. 4) | | | | | |
| --- | --- | --- | --- | --- | --- |
|  | *a* | *b* | *c* | | 100*.sd^a^* | |
|  | 0.9201 | -1.3073 | 141.812 | 0.41 | |
| Zafarani-Moattar et al. (Eq. 5) | | | | | |
|  | $\alpha$ | $\beta$ | $\gamma$ | 100*.sd* | |
|  | -0.2008 | -0.3869 | -1.2744 | 0.32 | |

*^a^* $sd=(\sum_{i=1}^{N} (w_{1}^{cal}-w_{1}^{\exp})^{2}/N)^{0.5}$where N and w_1_ represented number of binodal data and mass fraction of PEG_600_, respectively.

**Table S4**

The values of parameters of Hand, Bachman−Brown and Setschenow equations with the standard deviation of the models, *sd*, for the {PEG_600_ + KOH + H_2_O} at 298.15 K and atmospheric pressure (≈ 85 kPa)

| **Othmer-Tobias** | | |
| --- | --- | --- |
|  | | |
| *k* | *n* | *sd^a^* |
| 0.1505 | 1.1560 | 0.010 |
| **Bancraft** | | |
|  | | |
| *k_1_* | *r* | *sd* |
| 4.7985 | 0.648 | 0.013 |
| **Setschenow** | | |
|  | | |
| *K_p_* | *K_s_* | *sd* |
| 2.7368 | 11.0621 | 0.023 |

**Table S5**

The values of parameters of Eq. 13 and Eq. 14 along with the standard deviation of the models, *sd*, from the experimental values of partition coefficients for the {PEG_600_ + KOH + H_2_O} at 298.15 K and atmospheric pressure (≈ 85 kPa).

| Diamond-Hsu (Eq. 13) | | | | |  |
| --- | --- | --- | --- | --- | --- |
| *A* | 10^4^∙*B* | |  | | 100*.sd^a^* |
| Ibuprofen | | | | |  |
| 0.0437 | -0.2807 | |  | | 0.14 |
| Acetaminophen | | | |  | |
| 0.0254 | 2.1959 |  | | 0.15 | |
| Modified Diamond-Hsu (Eq. 14) | | | | | |
| *A*_1_ | *B*_1_ | | 10^3^∙*C*_1_ | 100.*sd* | |
| Ibuprofen | | | |  | |
| 4.3303 | -0.1088 | | 1.3067 | 0.06 | |
| Acetaminophen | | | |  | |
| 4.3303 | -0.1101 | | 1.4063 | 0.07 | |

*^a^* $sd=(\sum_{i=1}^{N} (K^{cal}-K^{\exp})^{2}/N)^{0.5}$ where $K$ and N represented the partition coefficients and number of partition coefficient data, respectively.

**Table S6**

The energy difference (**Δ***E_int_*) of Ibuprofen-PEG binary systems for the three functional methods.

| Binary system |  | | |
| --- | --- | --- | --- |
| Ibuprofen_PEG | B3LYP-D3(BJ) | M06-2x | WB97xd |
|  | -12.76 | -12.10 | -12.42 |
|  | -8.64 | -8.25 | -8.63 |
|  | -6.10 | -5.84 | -6.18 |
|  | -8.25 | -8.09 | -8.72 |
|  | -16.59 | -16.84 | -16.90 |
|  | -16.58 | -16.83 | -16.89 |

**Table S7**

The energy difference (**Δ***E_int_*) of Acetaminophen-PEG binary systems for the three functional methods.

| Binary system |  | | |
| --- | --- | --- | --- |
| Acetaminophen_PEG | B3LYP-D3(BJ) | M06-2x | WB97xd |
|  | -9.44 | -9.28 | -9.63 |
|  | -9.44 | -9.28 | -9.63 |
|  | -5.80 | -5.68 | -5.76 |
|  | -5.80 | -5.68 | -5.76 |
|  | -8.32 | -7.85 | -8.08 |
|  | -8.39 | -8.02 | -8.24 |
|  | -8.82 | -8.81 | -9.46 |
|  | -8.82 | -8.80 | -9.46 |
